# Supplementary figures and images for: Detours in long-distance migration across the Qinghai-Tibetan Plateau: individual consistency and habitat associations
Source: PeerJ. 2018 Jan 31;6:e4304. doi: 10.7717/peerj.4304 (PMC5797451; doi:10.7717/peerj.4304)

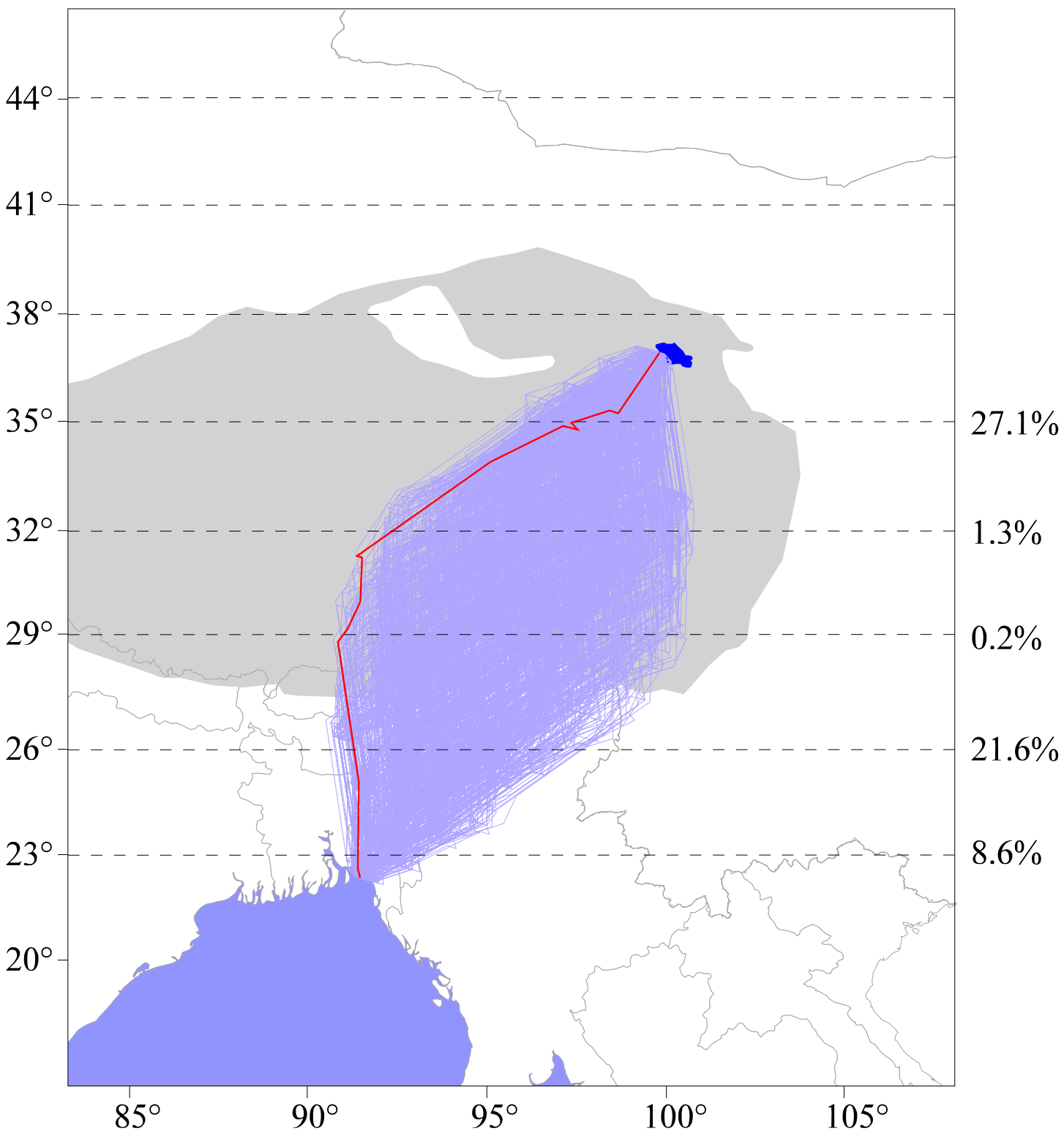

Supplement: Figure S1 — Spatial deviation in migration routes was analyzed by comparing observed route (red line) with 1,000 simulated routes (blue lines) obtained by randomly distributing the real route segments (see details in ‘Methods’). The number in the right column shows the percentile at which the longitude value of the observed route distributes in the 1000 longitude values of the simulated routes at each 3° latitude interval from 23°N to 44°N. If the percentile ia above or below 2.5% and 97.5%, the observed route was considered to deviate significantly from the simulated route at the specific latitude. [file peerj-06-4304-s002.pdf]

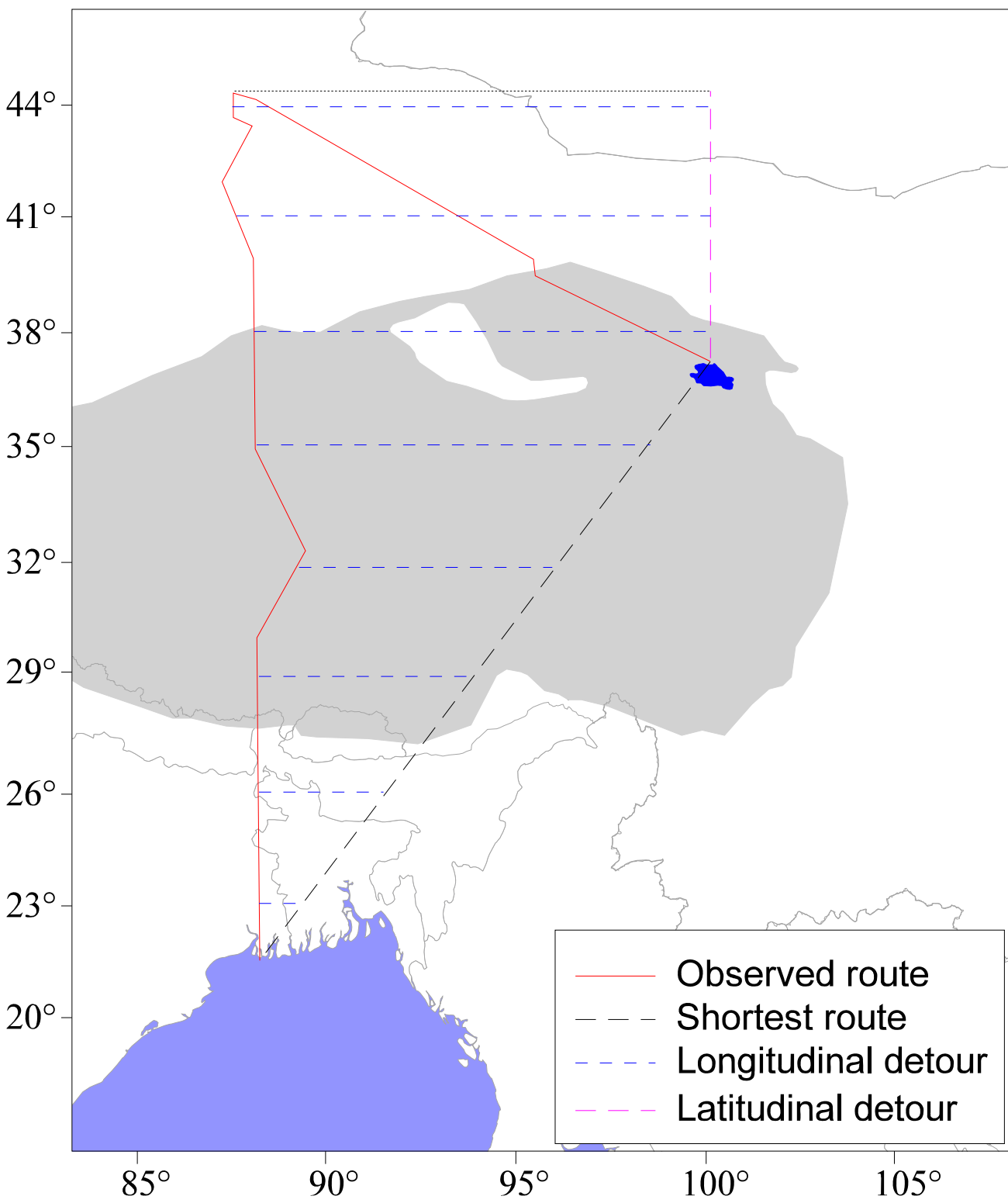

Supplement: Figure S2 [file peerj-06-4304-s003.pdf]
